# Supplementary material for: LjAMT2;2 Promotes Ammonium Nitrogen Transport during Arbuscular Mycorrhizal Fungi Symbiosis in Lotus japonicus
Source: Int J Mol Sci. 2022 Aug 23;23(17):9522. doi: 10.3390/ijms23179522 (PMC9455674; doi:10.3390/ijms23179522)
Supplement: Supplementary file 1 [file ijms-23-09522-s001.zip › ijms-1866782-supplementary.pdf]

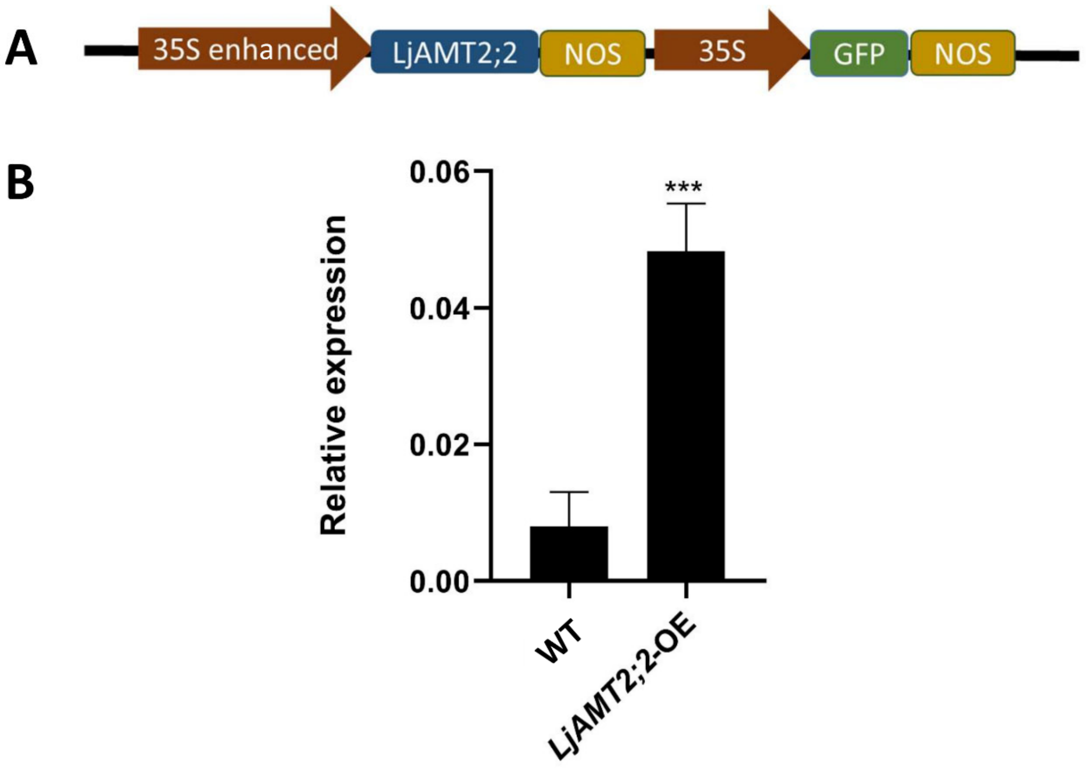

Figure S1. Construction of the *LjAMT2;2* overexpression vector and identification of *LjAMT2;2*-OE plants. (A) Construction of the *LjAMT2;2* overexpression vector. (B) Expression of the *LjAMT2;2* gene in *LjAMT2;2*-OE plants. *** $p \leq 0.001$.

Table S1. B&D culture medium.

Reagent	Concentration
$CaCl_2 \cdot 2H_2O$	1.0 M
K_2HPO_4	0.5 M
Fe-Citrate	0.01 M
$MgSO_4 \cdot 7H_2O$	0.25 M
K_2SO_4	1.5 M
KNO_3	1 M
$MnSO_4 \cdot H_2O$	10^{-3} M
H_3BO_3	2.0×10^{-3} M
$CuSO_4 \cdot 5H_2O$	2.0×10^{-4} M
$CoSO_4 \cdot 7H_2O$	10^{-4} M
$ZnSO_4 \cdot 7H_2O$	10^{-4} M
$NaMoO_4 \cdot 2H_2O$	5×10^{-4} M
